# New insights into chronic ankle instability: an *in vivo* evaluation of three-dimensional motion and stability of the ankle joint complex

**DOI:** 10.3389/fbioe.2025.1556291

**Published:** 2025-03-26

**Authors:** Shengli Wang, Yaokuan Ruan, Kaize Wang, Fei Chang, Boya Chen, Nan Zhang, Zhihui Qian, Lei Ren, Luquan Ren

**Affiliations:** ^1^ Key Laboratory of Bionic Engineering, Ministry of Education, Jilin University, Changchun, China; ^2^ Department of Orthopedics, The Second Hospital of Jilin University, Changchun, China; ^3^ Department of Radiology, The Second Hospital of Jilin University, Changchun, China; ^4^ Department of Mechanical, Aerospace and Civil Engineering, University of Manchester, Manchester, United Kingdom

**Keywords:** chronic ankle instability, ankle kinematics, biplanar radiography, ground reaction force, biomechanics

## Abstract

**Introduction:**

Chronic ankle instability (CAI) is generally associated with repetitive ankle sprains with concomitant ligament injuries and abnormal joint motion, which affects the stability of the joint. This study aims to quantify and compare the 3D motion differences in the ankle joint complex (AJC) during walking between CAI patients and healthy controls and to analyze the effect of CAI on the vertical ground reaction force (vGRF) and center of pressure (COP) distribution.

**Methods:**

Fifteen CAI patients (6 males, 9 females; height 165 ± 3.8 cm; weight 68.5 ± 10.2 kg; BMI 21.6 ± 3.5 kg/m^2^) with anterior talofibular and calcaneofibular ligament sprains and fifteen healthy participants (8 males, 7 females; height 168 ± 4.2 cm; weight 74.5 ± 12.6 kg; BMI 22.3 ± 4.2 kg/m^2^) participated in this study. Dynamic biplanar radiography were used to analyze the 3D motion and stability of the ankle joint complex during the stance phase. Synchronous force plate data were used to assess vGRF and COP trajectories.

**Results:**

Compared to controls, CAI patients showed increased plantarflexion (1.3°), internal rotation (2.0°), and medial translation (0.6 mm) in the tibiotalar joint, along with decreased dorsiflexion (3.0°). For the subtalar joint, plantarflexion decreased (1.8°), and external rotation increased (0.9°). The tibio-calcaneal joint showed increased internal rotation (1.9°) and posterior translation (0.5 mm). Stability differences included more dispersed axes of rotation and greater spatial motion volumes of landmarks in the CAI group. Additionally, CAI patients exhibited greater peak vGRF with earlier peaks, higher loading rates, and more lateral and unstable COP trajectories.

**Conclusion:**

These findings reveal that CAI not only alters the 3D motion and stability of the AJC but also affects foot-ground interaction forces, such as vGRF and COP distribution, during walking. This study provides critical insights into the altered biomechanics of the AJC in CAI patients and contributes to the clinical diagnosis of CAI and evaluation of results from surgical or conservative intervention.

## 1 Introduction

Lateral ankle sprains are among the most common musculoskeletal injuries, accounting for more than 80% of all ankle injuries (Fong et al., 2007; Caputo et al., 2009; Wikstrom et al., 2021; Yu et al., 2022). Lateral ankle sprains usually lead to lateral ligament injuries, resulting in stretching or rupturing of the anterior talofibular ligament (ATFL) and the calcaneofibular ligament (CFL) (Hass et al., 2010). While some patients recover well enough to resume normal activities after nonsurgical treatment, many others endure lingering symptoms such as pain, swelling, recurrent ankle sprains, and proprioceptive deficits accompanied by episodes of instability (Wikstrom et al., 2021; Ruan et al., 2024); this last symptom is termed chronic ankle instability (CAI), which can persist for months or even years following the initial sprain (Wikstrom and Brown, 2014; Koldenhoven et al., 2016; Han et al., 2022).

The ankle joint complex (AJC), comprising the tibiotalar and subtalar joints, plays a crucial role in maintaining stability and facilitating smooth locomotion (Roach et al., 2016; Yang et al., 2021; Wang et al., 2023; Wang et al., 2024). However, CAI significantly alters the biomechanics of these joints, thereby impairing their function (De Ridder et al., 2015; Herb et al., 2018). Many studies suggest that CAI alters the motion of the AJC in an abnormal manner, which may further lead to the development of degenerative changes, such as ankle arthritis (De Ridder et al., 2015; Hopkins et al., 2019; Jeon et al., 2021; Mousavi et al., 2023). Some researchers have also proposed that changes in joint movements play a role in the onset of osteoarthritis following lateral ligament injuries to the ankle (Hirose et al., 2004; Valderrabano et al., 2006). Therefore, precise measurement of the 3D motion of the AJC in patients with CAI during *in vivo* walking and comparison with healthy individuals are crucial. Early studies using optical motion capture and multisegmented foot models to analyze calcaneal motion relative to the leg have been insufficient for the precise quantification of 3D motion of the ankle joint in CAI patients (Delahunt et al., 2006; De Ridder et al., 2015; Wright et al., 2016; Yu et al., 2024). Dynamic biplanar radiography provides a new solution to this problem, but most of the recent studies have focused on quantifying 3D motion in healthy individuals during different activities (Roach et al., 2016; Pitcairn et al., 2020; Yang et al., 2021). Regarding the precise quantification or comparative analysis of the *in vivo* 3D motion of the ankle joint in CAI patients, in 2017, Roach et al. pioneered the use of a dual fluoroscopic system to study the kinematics of mechanical ankle instability during the early and late stance phases (Roach et al., 2017). They highlighted the importance of examining the ankle kinematics of CAI patients during dynamic activities. Unfortunately, their study did not report joint translations, and the gait was not continuous. Additionally, the sample size was small, and there was high variability in joint rotation among the four patients. The studies by Cao et al. on the 3D motion of the AJC in patients with functional ankle instability and by Caputo et al. on tibiotalar kinematics in patients with lateral ankle instability associated with ATFL injury have provided valuable data for the characterization of ankle motion in CAI patients (Caputo et al., 2009; Cao et al., 2019). However, these studies may have been conducted under conditions of non-continuous locomotion or low-frequency acquisition using with key postures and quasistatic weight-bearing postures and therefore may lack quantitative analysis of continuous walking data. Therefore, quantitative analysis of the 3D motion of the AJC in patients with CAI during continuous walking and comparative analysis with healthy individuals are still inadequate. Additionally, there is still a lack of studies quantifying the differences in joint stability between CAI patients and healthy individuals from the perspective of joint motion.

**FIGURE 1:**
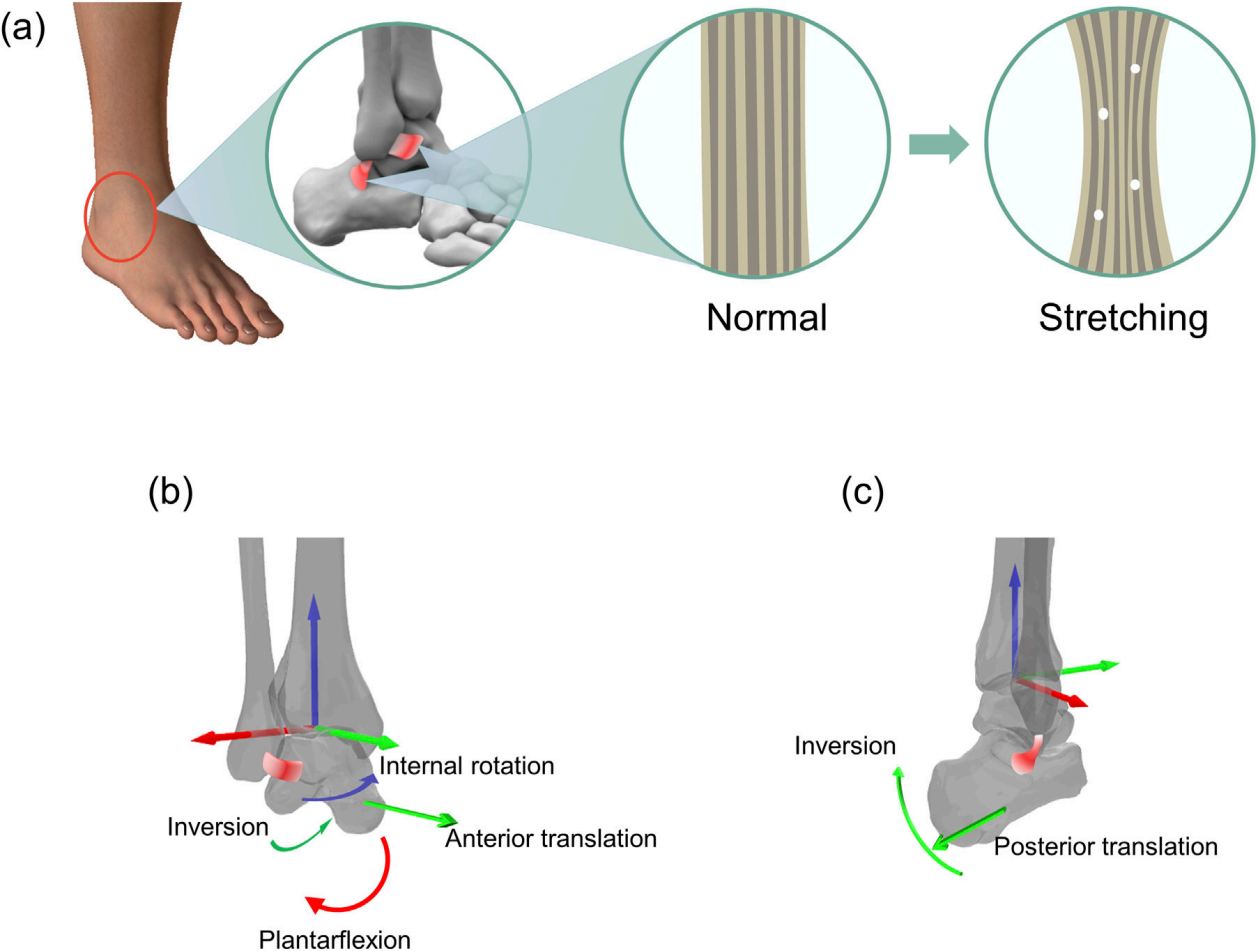
Schematic representation of an overstretched anterior talofibular ligament (ATFL) and calcaneofibular ligament (CFL) and the resulting (hypothetical) altered motion of the corresponding joints. **(a)** Morphological changes corresponding to the stretching of the ATFL and CFL. **(b)** Stretching of the ATFL and the hypothetical resulting altered motion of the tibiotalar joint. **(c)** Stretching of the CFL and the hypothetical resulting altered motion of the tibio-calcaneal joint.

Therefore, this study aimed to quantitatively analyze the 3D motion and stability of the AJC during walking in CAI patients (specifically those with ATFL and CFL injuries) (Figure 1a) and to compare the results with those of healthy individuals, thereby addressing the current gaps in knowledge. Based on the orientation of the ATFL at the tibiotalar joint, it was hypothesized that CAI patients would exhibit more plantarflexion, inversion, internal rotation, and anterior translation (talus relative to the tibia) at the tibiotalar joint (Figure 1b). According to the orientation of the CFL at the tibio-calcaneal joint, it was hypothesized that CAI patients would show increased inversion and posterior translation (calcaneus relative to the tibia) (Figure 1c). In addition, walking, as a fundamental human activity, relies on the intricate coordination of various joints and muscles to maintain balance and propel the body forward. In individuals with CAI, the altered mechanics of the AJC can lead to deviations in walking patterns, increased instability, and a greater susceptibility to recurrent injuries. Therefore, it was reasoned that CAI patients experience alterations in not only the 3D motion and stability of the local joint (i.e., the AJC) but also altered interaction forces between the foot and the ground; therefore, the magnitude and loading of the vertical ground reaction force (vGRF) and the distribution of the center of pressure (COP) trajectory were also simultaneously analyzed. Through this approach, new insights were gained into the motion and stability of the AJC and the accompanying foot-ground interaction forces in CAI patients. This will provide effective comparative data for the clinical diagnosis of CAI and the assessment of postoperative rehabilitation.

## 2 Materials and methods

### 2.1 Participant recruitment

A power analysis was conducted using the SamplePower module in SPSS software to determine the minimum sample size required. The estimated effect size was set to 0.80 (Cohen’s d), which represents a large effect size as per Cohen’s criteria. The results of the analysis showed that the sample size of 15 participants per group met the statistical requirements with a significance level of 0.05 and power of 0.80. After obtaining approval from the Ethics Committee of the Second Hospital of Jilin University (No. 2023080), 15 patients (6 males, 9 females) and 15 healthy participants (8 males, 7 females) provided informed consent and voluntarily participated in this study. All participants signed written informed consent forms before the start of the experiment. All participants were between 25 and 45 years of age. Detailed demographic characteristics and radiographic grading of ligamentous injury are provided in Supplementary Table S1 of the Supplementary Material. The patients involved in this study were chosen from those attending the foot and ankle surgery clinic for CAI treatment. All patients were examined and diagnosed by specially trained and experienced foot and ankle surgeons.

The inclusion criteria for CAI patients were as follows (Ruan et al., 2024): (1) history of unilateral ankle sprain based on a questionnaire, more than 1 year since the first injury, and significant sensation of instability; (2) at least 3 instances of recurrent ankle sprains; (3) positive results on the talus tilt test and the anterior drawer test; and (4) Magnetic resonance imaging in the last week showed ATFL and CFL sprains, with or without posterior talofibular ligament (PTFL) sprains. The exclusion criteria were as follows: (1) unilateral or bilateral ankle arthritis; and (2) a history of significant trauma or surgery on either lower limb. Through the survey questionnaire, it was determined that none of the healthy control participants had a history of ankle sprains or a sensation of ankle instability. Magnetic resonance imaging revealed that the control group participants had intact ATFL, PTFL, and CFL.

### 2.2 Data collection

**FIGURE 2:**
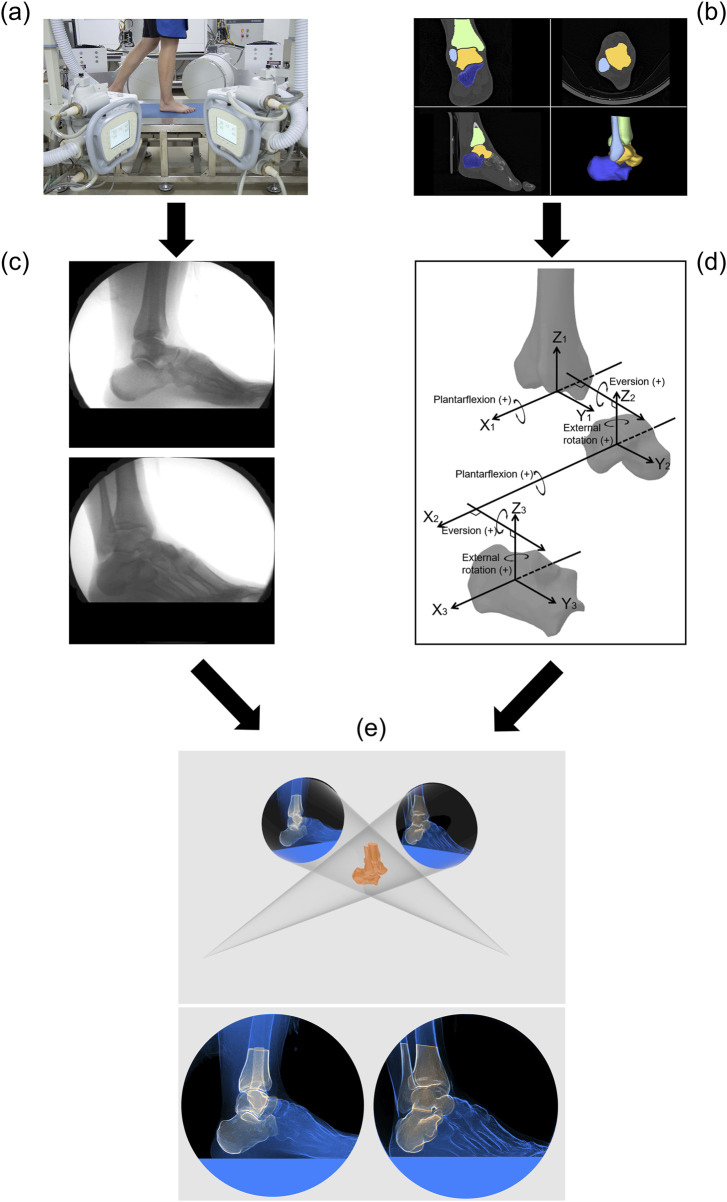
Flowchart of the experimental methods and techniques. **(a)** Collection of biplanar radiograph sequence during the stance phase of gait. **(b)** CT image acquisition and segmentation. **(c)** Biplanar radiograph sequence. **(d)** Anatomical coordinate systems for the tibia, talus, and calcaneus and joint coordinate systems for the tibiotalar and subtalar joints. **(e)** Model-based 2D-3D dynamic tracking.

The biplanar radiography system (Imaging Systems & Service, Painesville, United States) consisted of two X-ray tubes and two image intensifiers capable of acquiring X-ray image sequences from two directions simultaneously. A 5.6-m-long walkway was laid between the tubes and the intensifiers, with a force plate (BMS400600, AMTI, United States) embedded in the middle of the walkway (Figure 2a). The central position of the force plate roughly corresponded to the image acquisition area. Before image acquisition, participants were given sufficient time to practice walking until their target foot reliably landed in the designated area. Each participant walked at their preferred natural speed, and biplanar radiograph sequences were captured during their walk (Figure 2c). The laboratory is equipped with a high-speed camera (FDRAX700, Sony, Japan) to capture gait, and the calculated walking speeds in the narrow range of 1.25 m/s to 1.76 m/s (mean ± SD: 1.45 ± 0.27 m/s). A minimum of 5 trials and valid data were collected for each participant. The images were acquired at a frequency of 120 Hz, with a voltage of 58 kV, a current of 80 mA, and an image resolution of 1,024 × 1,024 pixels. The biplanar radiography system and the force plate shared a synchronous trigger device, allowing the simultaneous acquisition of GRF data (1,000 Hz) during image capture. A threshold of 30 N was set for the vGRF to determine heel strike and toe-off events (Yang et al., 2021; Wang et al., 2024).

CT images of the CAI patients were obtained from clinical examinations, and all images included the complete AJC. CT images of the healthy participants were acquired using the same CT scanner (Brilliance iCT 256, Philips, Netherlands) used for the patients. Acquisition parameters: slice thickness, 0.8 mm; average resolution, 0.578 × 0.578 × 0.400 mm; image matrix, 512 × 512 pixels. The medical image processing software OsiriX (Pixmeo, Geneva, Switzerland) was used for CT image segmentation and model reconstruction (Camp and Brainerd, 2014; Kambic et al., 2014; Camp et al., 2015). First, the cross-sectional images were segmented to include only the region of interest (i.e., only the target bones) (Figure 2b), and an image stack was synthesized to subsequently generate a virtual model for registration. The 3D polygonal mesh model of the AJC was reconstructed from the region of interest of the segmented CT images to identify anatomical bony landmarks and define the anatomical coordinate system. A MATLAB-based toolbox called AAFACT, previously reported by Peterson et al., was used to create an anatomical coordinate system for each bone (Figure 2d) (Peterson et al., 2023). This toolbox automatically calculated the anatomical coordinate systems of the bones with good robustness and consistency (Peterson et al., 2023; Knutson et al., 2024).

### 2.3 Image processing

The motion data of the bones in 3D space were obtained using model-based 2D-3D registration technology. Model-based tracking was performed using a previously published method with Autoscoper software (V2, Brown University) (Miranda et al., 2011; Kessler et al., 2019; Maharaj et al., 2020). The CT images containing only the region of interest were synthesized into an image stack to generate digitally reconstructed radiographs (DRRs) in a virtual 3D space. The DRRs were manually aligned with the bone contours in the two X-ray images through rotation and translation. Then, particle swarm optimization was used to perform automatic tracking until alignment was completed for every frame in the image sequence (Figure 2e). Finally, the pose matrix corresponding to each bone was obtained for each frame of the stance phase. The accuracy of model-based tracking was 0.71° ± 0.12° for rotation and 0.59 ± 0.10 mm for translation (You et al., 2001; Bey et al., 2006; Cross et al., 2017).

### 2.4 Data analysis

The 3D kinematics of the three joints were calculated using the joint coordinate system proposed by Grood et al. (Figure 2d) (Grood and Suntay, 1983; Yang et al., 2021). The kinematic and ground reaction force data were filtered by a 4th order low-pass Butterworth filter with cutoff frequencies of 10 Hz and 60 Hz, respectively. The stance phase was normalized, with heel strike corresponding to 0% and toe-off corresponding to 100%.

The instantaneous axes of rotation of the joints were determined using a method of fitting the geometric surfaces of the bones; specifically, the axis of the tibiotalar joint passes through the centers of the fitted spheres of the medial and lateral trochlear facets of the talus, while the axis of the subtalar joint passes through the centers of the fitted spheres of the medial and posterior facets of the calcaneus (Parr et al., 2012; Nichols et al., 2017; Claassen et al., 2019; Yu et al., 2023). The positions of the instantaneous axes of rotation are expressed in the local coordinate system of the proximal bone, and their discrete distribution characteristics can reflect the stability of the joint to some extent (Temporiti et al., 2020; Ancillao, 2022). Joint stability was quantified in two ways (Wang et al., 2024): the stability of the tibiotalar joint was assessed by comparing the distribution area of the intersection of the tibiotalar joint axis with the sagittal plane and the space volume of motion of the anterior and posterior talus prominences, and the stability of the subtalar joint was assessed by comparing the distribution area of the intersection of the subtalar joint axis with the transverse plane and the space volume of motion of the anterior and posterior calcaneal prominences. The larger the area or volume was, the more unstable the joint.

All vGRF values were normalized to each participant’s body weight. During the initial loading phase, the ratio of the force increase to the corresponding time was defined as the loading rate (Kluitenberg et al., 2012; Bigouette et al., 2016). The position of the COP trajectory on the force plate plane was calculated based on the relationship between the force and moment arm. The last posterior point of each trajectory was aligned to the origin, and the length (anterior-posterior direction) was normalized as a percentage of the maximum length.

### 2.5 Statistical analysis

Statistical differences in continuous kinematic waveforms between the two groups were assessed using one-dimensional statistical parametric mapping (Pataky, 2010; Yang et al., 2021). Unpaired Student's t tests were used to identify differences in the range of motion (ROM) of the six degrees of freedom during the stance phase of walking. The effective distribution area of the intersections between the instantaneous axis of rotation and the anatomical plane was expressed using a 95% confidence ellipse, while the effective spatial volume of anatomical landmarks in the local coordinate system was expressed using a 95% confidence ellipsoid; significant differences between the two groups were identified using unpaired Student's t tests. All statistical differences were considered significant when P was less than 0.05.

## 3 Results

### 3.1 Continuous 3D motion waveforms

**FIGURE 3:**
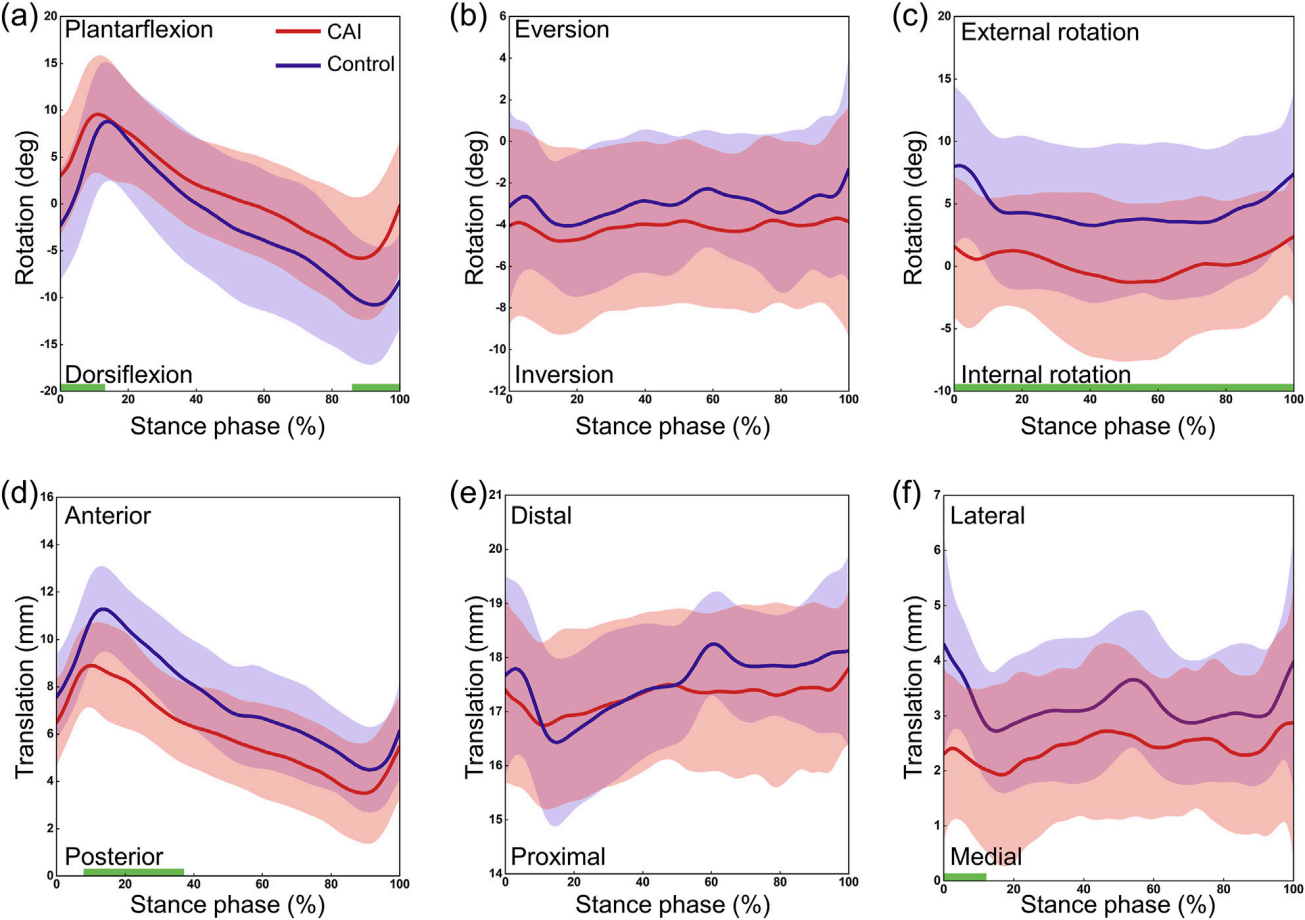
3D kinematics of the tibiotalar joint during the stance phase of gait in the CAI group (red) and the control group (blue). **(a)** Dorsiflexion/Plantarflexion. **(b)** Eversion/Inversion. **(c)** External/Internal Rotation. **(d)** Anterior/Posterior Translation. **(e)** Proximal/Distal Translation. **(f)** Lateral/Medial Translation. The shaded areas represent ±1 standard deviation. The intervals with significant differences are marked with a thick green line under each subplot.

**FIGURE 4:**
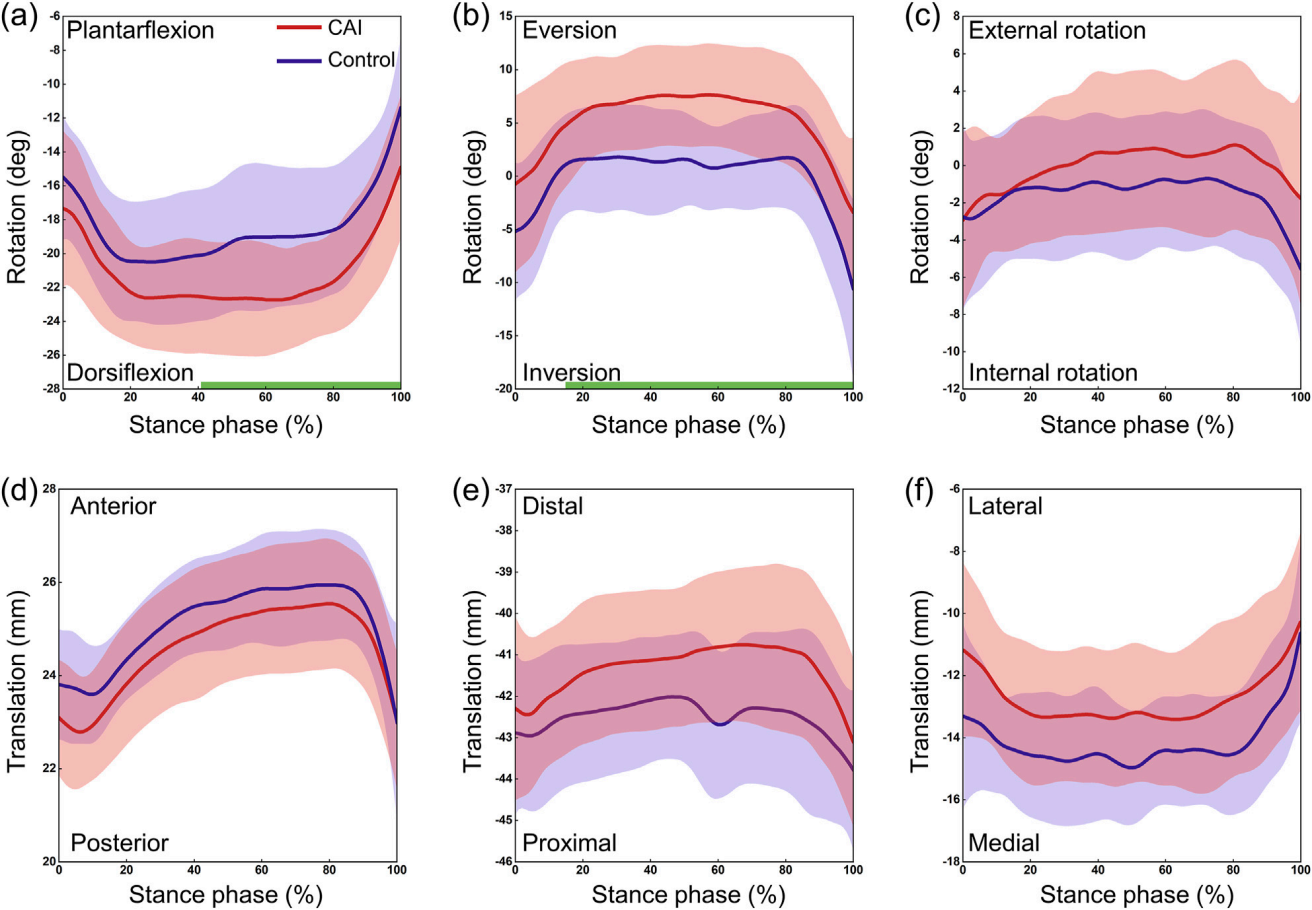
3D kinematics of the subtalar joint during the stance phase of gait in the CAI group (red) and the control group (blue). **(a)** Dorsiflexion/Plantarflexion. **(b)** Eversion/Inversion. **(c)** External/Internal Rotation. **(d)** Anterior/Posterior Translation. **(e)** Proximal/Distal Translation. **(f)** Lateral/Medial Translation. The shaded areas represent ±1 standard deviation. The intervals with significant differences are marked with a thick green line under each subplot.

The CAI group and the control group exhibited similar continuous kinematic waveforms, but there were noticeable differences in multiple degrees of freedom (Figure 3). At the tibiotalar joint, the CAI group exhibited greater plantarflexion during the early (0%–11%) and late (88%–100%) stance phases (P = 0.038 and P = 0.012, respectively) and more internal rotation during the stance phase (P = 0.017). The talus showed greater posterior translation relative to the tibia during the post-heel strike phase (10%–35%) (P = 0.002) and more medial translation during the initial phase (0%–10%) (P = 0.011). At the subtalar joint, the CAI group demonstrated greater dorsiflexion during the 43%–100% stance phase (P < 0.001) and more eversion during the 17%–100% stance phase (P < 0.001) (Figure 4). The mean curve shows that the calcaneus is more posterior, distal and lateral to the talus in patients with CAI. At the tibio-calcaneal joint, the CAI group exhibited greater plantarflexion during the early (0%–5%) and late (93%–100%) stance phases (P = 0.027 and P = 0.026, respectively) and greater internal rotation during the initial stance phase (0%–8%) (P = 0.039) (Supplementary Figure S1, Supplementary Material). Specifically, the internal/external rotation (IR/ER) of the CAI and control groups showed opposite trends at the beginning of the stance phase. Throughout almost the entire stance phase, the calcaneus was positioned more posteriorly and laterally relative to the tibia (all P < 0.001).

### 3.2 ROM in 6 degrees of freedom

**FIGURE 5:**
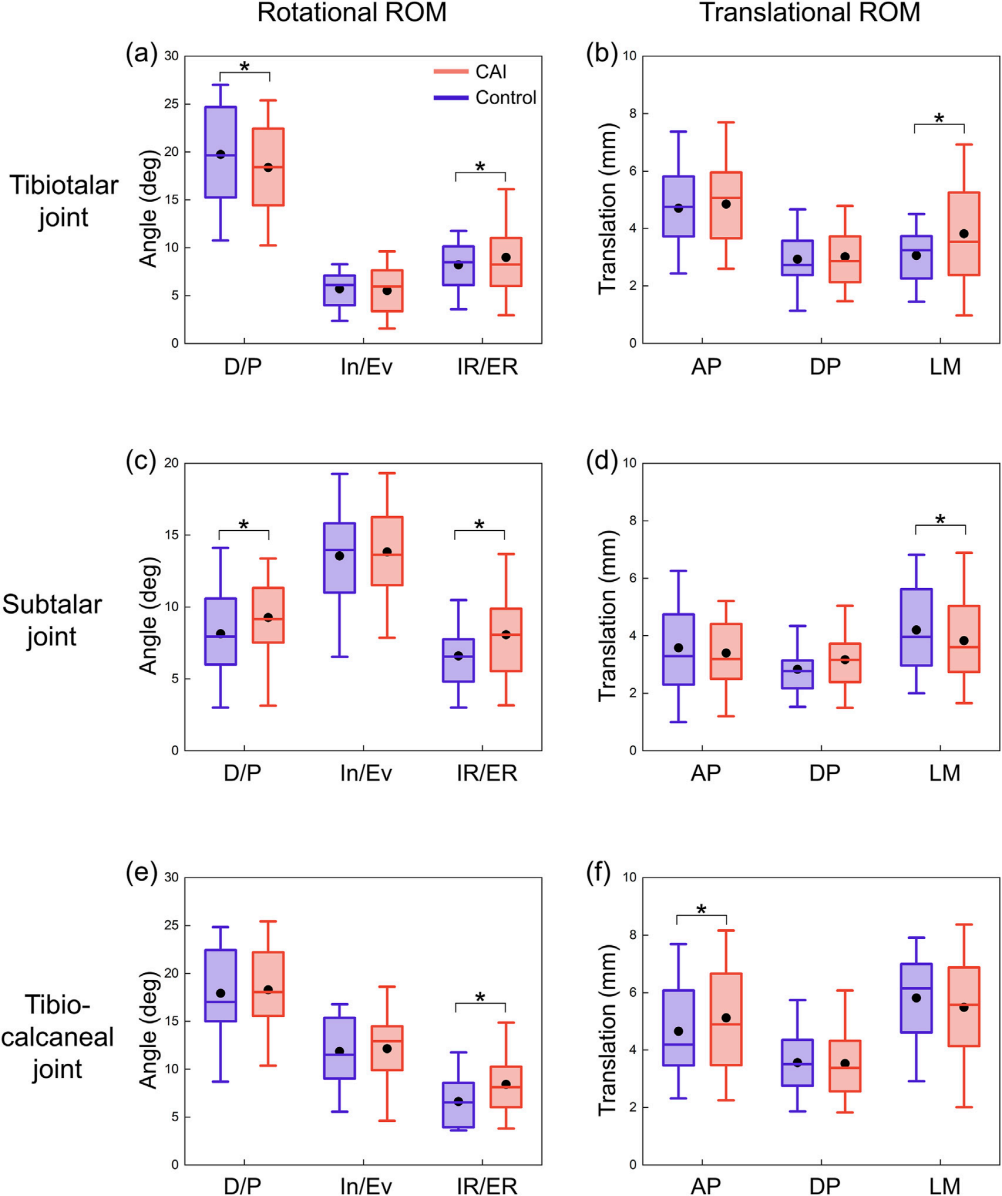
ROMs of the tibiotalar, subtalar, and tibio-calcaneal joints during the stance phase for CAI patients and control group. **(a)** Rotational ROM of the tibiotalar joint. **(b)** Translational ROM of the tibiotalar joint. **(c)** Rotational ROM of the subtalar joint. **(d)** Translational ROM of the subtalar joint. **(e)** Rotational ROM of the tibio-calcaneal joint. **(f)** Translational ROM of the tibio-calcaneal joint. The black dots represent mean values. D/P: Dorsiflexion/Plantarflexion; In/Ev: Inversion/Eversion; IR/ER: Internal/External Rotation; AP: Anterior-Posterior; DP: Distal-Proximal; LM: Lateral-Medial. *P < 0.05.

For the tibiotalar joint, plantarflexion ROM increased by 1.3° and dorsiflexion ROM decreased by 3.0° in the CAI group, and total dorsiflexion/plantarflexion (D/P) ROM decreased significantly (Table 1; Figure 5a). In addition, internal rotation ROM increased by 2.0° and medial translation increased by 0.6 mm. For the subtalar joint, the CAI group decreased the ROM for plantarflexion by 1.8° and increased the external rotation ROM by 0.9° (Table 1). D/P and IR/ER ROM both increased significantly, while LM translation ROM decreased significantly (Figures 5c, d). For the tibio-calcaneal joint, the CAI group increased ROM by 1.9° of internal rotation and 0.5 mm of posterior translation, with a significant increase in IR/ER ROM and anterior-posterior (AP) translation ROM (all P < 0.05).

**TABLE 1:** The range of motion (ROM) comparison data for each direction between the CAI group and the control group, with the neutral position as the reference. *P < 0.05.

3D motion	Tibiotalar joint		Subtalar joint		Tibio-calcaneal joint	
	CAI	Control	CAI	Control	CAI	Control
Plantarflexion (°)	6.2 ± 3.1*	4.9 ± 2.3*	8.0 ± 2.8*	9.8 ± 4.5*	8.8 ± 2.4	7.9 ± 2.0
Dorsiflexion (°)	11.4 ± 4.9*	14.4 ± 4.4*	1.1 ± 0.8	1.0 ± 0.7	9.2 ± 3.7	10.3 ± 5.2
Eversion (°)	2.9 ± 2.2	3.5 ± 2.8	1.9 ± 1.4	2.0 ± 1.6	2.3 ± 1.6	2.0 ± 1.7
Inversion (°)	3.1 ± 2.5	2.7 ± 2.4	11.8 ± 3.4	13.3 ± 5.6	10.3 ± 3.5	10.7 ± 5.1
External rotation (°)	4.9 ± 4.7*	6.0 ± 4.1*	3.3 ± 2.5*	2.4 ± 1.7*	3.5 ± 3.2	3.9 ± 2.0
Internal rotation (°)	4.6 ± 3.3*	2.6 ± 2.2*	5.1 ± 2.8	5.5 ± 3.7	5.0 ± 3.3*	3.1 ± 2.7*
Anterior (mm)	2.4 ± 1.2	2.3 ± 0.9	2.0 ± 1.5	2.7 ± 1.5	0.8 ± 0.7	0.6 ± 0.2
Posterior (mm)	3.1 ± 1.7	3.4 ± 2.4	2.5 ± 2.0	2.9 ± 3.1	4.1 ± 1.4*	3.6 ± 2.5*
Proximal (mm)	1.0 ± 0.7	1.0 ± 0.4	2.3 ± 1.1	2.0 ± 0.8	2.9 ± 1.0	3.1 ± 1.0
Distal (mm)	1.2 ± 0.8	2.0 ± 1.1	1.0 ± 0.6	0.8 ± 0.4	0.6 ± 0.6	0.4 ± 0.3
Lateral (mm)	2.2 ± 1.3	2.4 ± 1.4	3.4 ± 3.0	3.7 ± 2.7	4.5 ± 1.9	5.6 ± 2.1
Medial (mm)	1.7 ± 1.2*	1.1 ± 0.7*	1.6 ± 1.7	1.5 ± 1.4	1.0 ± 0.8	0.9 ± 0.5

### 3.3 Instantaneous axis of rotation and joint stability

**FIGURE 6:**
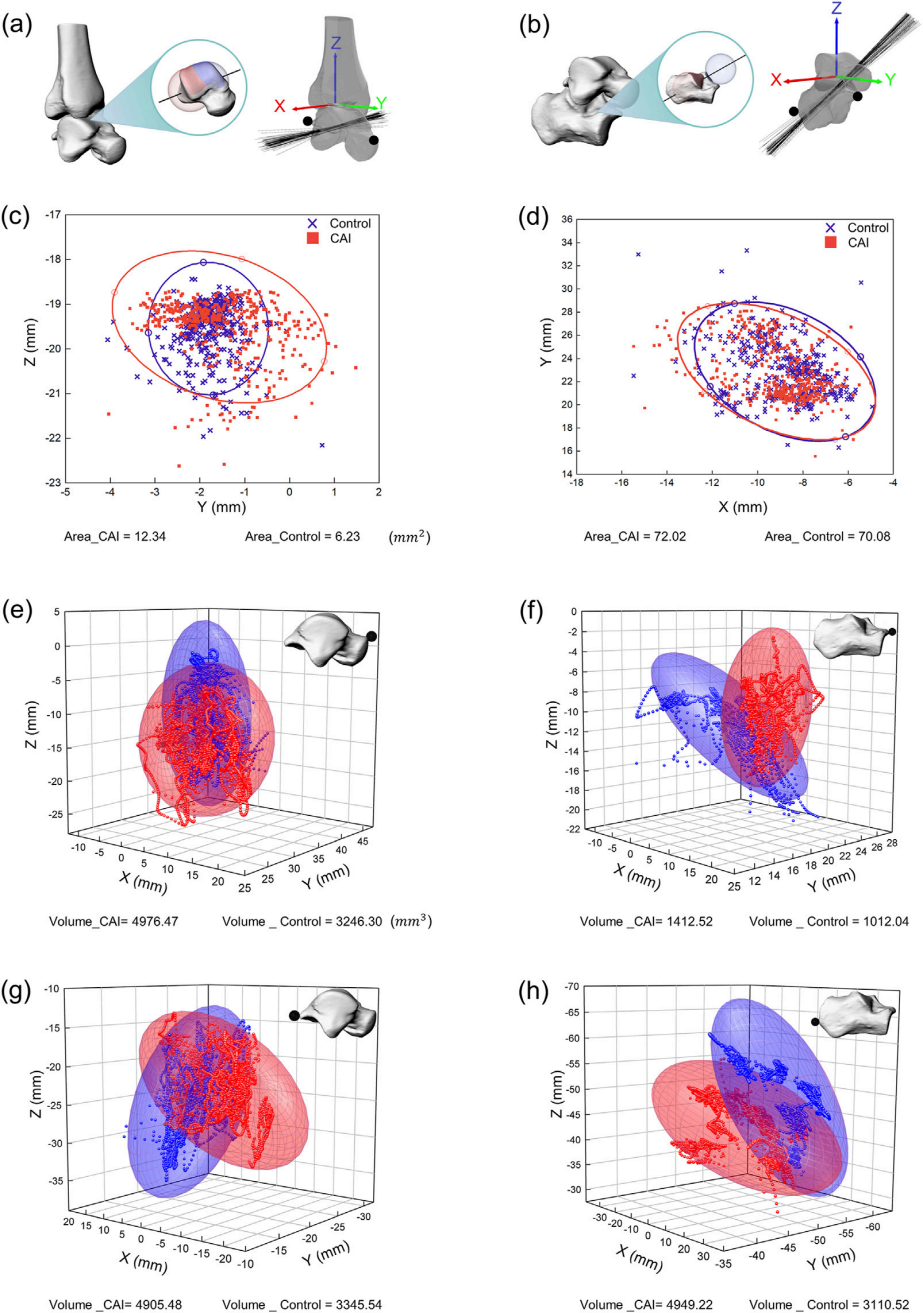
Spatial representation of the instantaneous axes of rotation and the motion volume of specified anatomical landmarks for all participants. **(a)** Instantaneous axis of rotation for the tibiotalar joint. **(b)** Instantaneous axis of rotation for the subtalar joint. **(c)** Intersection of the instantaneous axis of rotation of the tibiotalar joint with the sagittal plane (YZ plane). **(d)** Intersection of the instantaneous axis of rotation of the subtalar joint with the transverse plane (XY plane). The distribution of intersections between the instantaneous axes and anatomical planes is represented by 95% confidence ellipses (red ellipse for CAI patients, blue ellipse for the control group). **(e)** Distribution of the anterior prominence of the talus in 3D space. **(f)** Distribution of the anterior prominence of the calcaneus in 3D space. **(g)** Distribution of the posterior prominence of the talus in 3D space. **(h)** Distribution of the posterior prominence of the calcaneus in 3D space. The motion volume of specified anatomical landmarks in the proximal coordinate system is represented by 95% confidence ellipsoids (red ellipsoid for CAI patients, blue ellipsoid for the control group).

The area of the 95% confidence ellipse for the distribution of the intersection between the axis of the tibiotalar joint and the sagittal plane was significantly larger in the CAI group (P < 0.001), at 12.34 mm^2^ for all participants in the CAI group compared with 6.23 mm^2^ for all participants in the control group (Figure 6c). The area of the 95% confidence ellipse distributed at the intersection of the axis of the subtalar joint and the transverse plane was not significantly different between the two groups (72.02 mm^2^ and 70.08 mm^2^, respectively) (Figure 6d).

The motion space of the anterior and posterior prominences of the talus in the local coordinate system was significantly greater in the CAI group (all P < 0.05). The 95% confidence ellipsoid volumes for the anterior prominences of the talus (for all participants in all trials) were 4,976.47 mm^3^ for the CAI group and 3,246.30 mm^3^ for the control group (Figure 6e). For the posterior prominences of the talus, the volumes were 4,905.48 mm^3^ and 3,345.54 mm^3^, respectively (Figure 6g). The motion space of the anterior prominences of the calcaneus in the local coordinate system was significantly greater in the CAI group than in the control group (P = 0.012), with volumes of 1,412.52 mm^3^ and 1,012.04 mm^3^, respectively (Figure 6F). For the posterior prominences of the calcaneus, the volumes were 4,949.22 mm^3^ and 3,110.52 mm^3^, respectively (Figure 6h), but the difference between the two groups was not significant.

### 3.4 vGRF and COP

**FIGURE 7:**
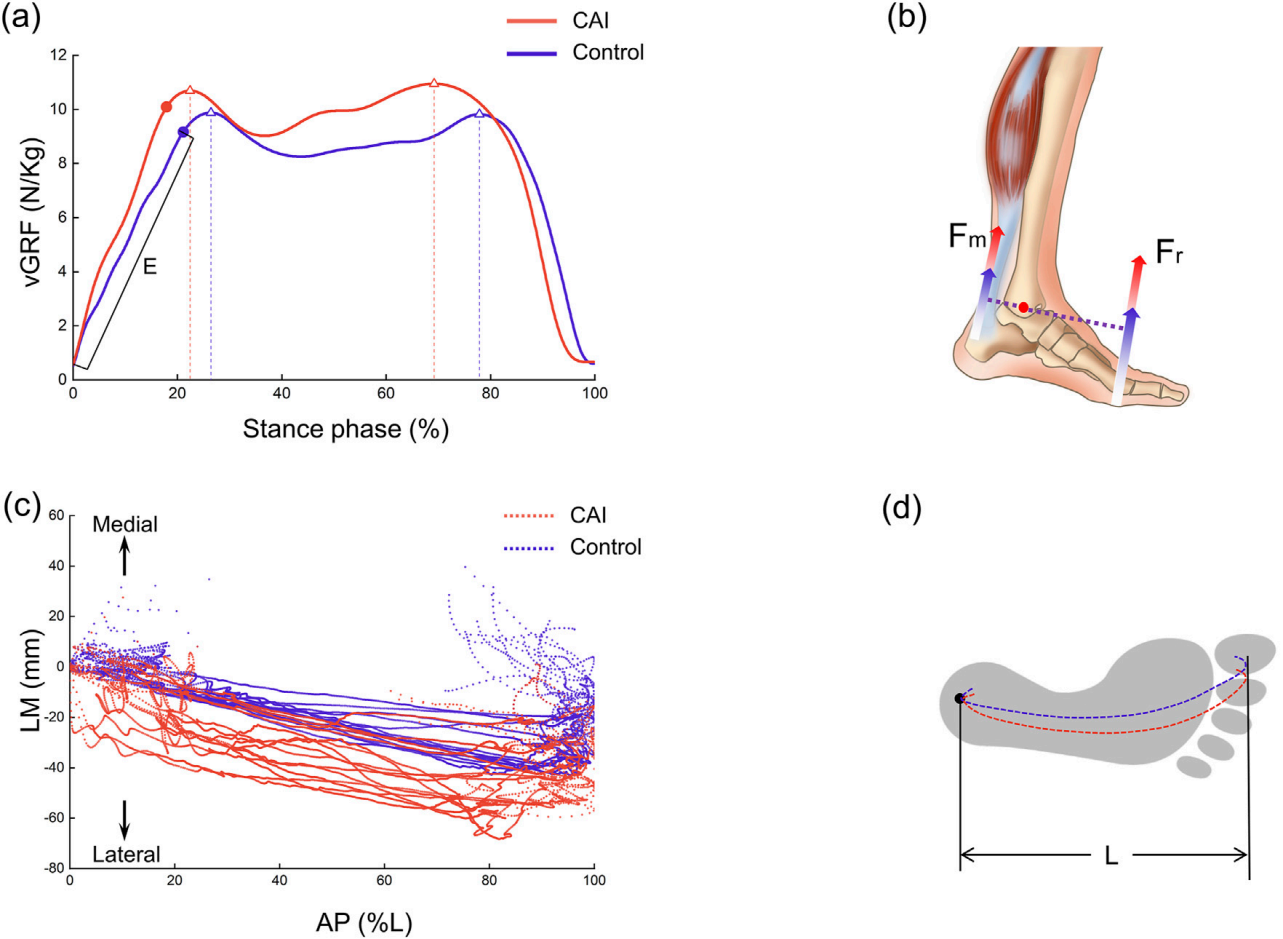
Vertical ground reaction force (vGRF) and center of pressure (COP) trajectories during the stance phase. **(a)** Variation curve and peak values of vGRF (indicated by triangles) and loading rate E (N/kg/s). **(b)** Increased vGRF was inferred to increase the force of plantarflexor. **(c)** Representation of the position of the COP trajectory on the force plate plane. LM: lateral-medial, AP: anterior-posterior. **(d)** Schematic diagram of COP trajectory positions within the footprint.

The CAI group demonstrated a greater peak vGRF (P = 0.004) and a shorter time to peak (P = 0.036) than did the control group (Figure 7a). The first peak increased by approximately 0.85 N/kg, and the second peak increased by approximately 1.13 N/kg. Specifically, the first vGRF peak values for the CAI group and the healthy group were 759.73 ± 40.37 N and 692.80 ± 34.72 N, respectively, and the second vGRF peak values were 767.60 ± 45.52 N and 650.86 ± 46.51 N, respectively. Additionally, the loading rate was greater in the CAI group (P = 0.025). The trajectory of the COP was more unstable in the CAI group, with 11 participants showing greater lateral deviation, while 4 participants had trajectories within the coverage area of the control group’s COP (Figure 7c).

## 4 Discussion

This study quantified the 3D motion of the AJC using a biplanar fluoroscopy system and model-based 2D-3D tracking techniques, comparing CAI patients with a healthy control group. Additionally, comparisons were made between the CAI patients and healthy controls regarding vGRF and COP trajectories. Compared to the control group, CAI patients exhibited increased dorsiflexion, internal rotation, and medial translation in the tibiotalar joint. There was a decrease in dorsiflexion and an increase in external rotation at the subtalar joint. Increased internal rotation and posterior translation were noted in the tibio-calcaneal joint. The rotational axis in the CAI group was more dispersed, and there was greater spatial motion of anatomical landmarks. Furthermore, CAI patients exhibited higher peak vGRF, earlier peak times, higher loading rates, and more laterally and unstable COP trajectories. Thus, this study provides new insights into CAI from both kinematic and foot-ground interaction mechanical perspectives.

This study revealed that the kinematic waveforms of the AJC in both CAI patients and controls followed a similar pattern. However, there were still some differences between the two groups. Specifically, the tibiotalar joint in the CAI group exhibited significant plantarflexion and internal rotation during the stance phase, and the tibio-calcaneal joint exhibited significant posterior translation, which is consistent with our hypothesis. The 3D continuous motion of the tibiotalar and subtalar joints in healthy individuals has been reported in the literature, and the results of the present study are similar to these reports (Koo et al., 2015; Roach et al., 2016; Yang et al., 2021). Specifically, the motion waveforms reported in this study are highly similar to those in the study by Yang et al. because the same kinematic computation method was used (Yang et al., 2021), although there was a slight difference in the definition of the local coordinate system. There are very few studies on the use of biplane dynamic fluoroscopy to study the 3D continuous motion of the AJC in patients with CAI. The only available study is by Roach et al., who investigated the rotational motion of the ankle joint in four CAI patients during the beginning and end of the stance phase (Roach et al., 2017). The relatively small number of participants, as well as the large variability and the incompleteness of the stance phase, prevent a direct comparison with this study. In a quasistatic weight-bearing motion test, Caputo et al. noted significant internal rotation of the talus in individuals with ATFL injuries and suggested that excessive internal rotation increases medial cartilage wear in the tibiotalar joint, increasing susceptibility to ankle arthritis (Caputo et al., 2009). The results of this study also indicated increased internal rotation of the tibiotalar joint in patients with CAI (Figure 3c; Table 1). However, the motion in other directions differed from that reported by Caputo et al., primarily due to the different motion patterns used in the test. This study represents a significant advancement based on these previous findings. For the 3D kinematics of the AJC during weight-bearing walking in patients with CAI, a more comprehensive description of the continuous kinematic waveforms and ROM for the tibiotalar, subtalar, and tibio-calcaneal joints was provided.

After initial contact (10%–35% phase of gait), the talus showed significant posterior displacement relative to the tibia in the CAI group compared to the control group (Figure 3d), which contradicts our hypothesis. It has been hypothesized that this may be related to a larger GRF on the side affected by the CAI, but this study lacks corresponding modeling or theoretical validation and is only speculative for the following reasons. The results suggested that the vGRF was greater in CAI patients than in healthy individuals, with the first and second peaks increasing by approximately 0.85 N/kg and 1.13 N/kg, respectively (Figure 7a). In fact, the resultant force (Fr) in the three directions was also greater in the CAI group, and at initial contact, the Fr was toward the posterior-superior direction, with a backward component. This backward component was also greater in CAI patients than in healthy individuals, leading to a posterior shift of the talus due to the drawer-like motion of the ankle joint in the AP direction. This may be one of the reasons leading to PTFL and CFL injuries. CAI does not significantly affect the inversion/eversion (In/Ev) of the tibiotalar or tibio-calcaneal joints, as neither the continuous kinematic waveforms nor the ROM measurements supported the hypotheses regarding the effect of these ligament injuries on the In/Ev of the tibiotalar or tibio-calcaneal joints. This study provides a detailed report on the differences in subtalar joint motion between the two groups. The direction of deviation of the subtalar joint appears to be opposite to that of the tibiotalar joint. Specifically, the mean curves indicated that the tibiotalar joint in the CAI group exhibited more plantarflexion (Figure 3a), inversion (Figure 3b), and internal rotation (Figure 3c), while the subtalar joint showed more dorsiflexion (Figure 4a), eversion (Figure 4b), and external rotation (Figure 4c). Considering the influence of walking speed on ankle joint kinematics, statistical analysis has shown that within a narrow range of walking speeds (1.25 m/s to 1.76 m/s), there is no significant correlation between the kinematic data (ROM) of the AJC and walking speed (Pearson correlation coefficient r < 0.3, P > 0.05). This eliminates the impact of walking speed on the kinematics of the ankle joint.

Injury to the ATFL had a more pronounced effect on motion in the LM direction of the tibiotalar joint, as patients with CAI had a significantly greater ROM for IR/ER and LM translation than did controls (Figures 5a, b). The injured ligaments became lax, decreasing the constraints on the medially oriented motion of the bones. The dominant influence of increased internal rotation of the talus may partially explain why CAI patients are more prone to medial cartilage damage in the tibiotalar joint, potentially leading to ankle osteoarthritis.

In this study, the stability of the AJC in CAI patients was quantified and compared with those in healthy controls, which has not been reported in the literature. From the perspectives of the discrete distribution area of the instantaneous rotation axis and the spatial motion volume of specified anatomical landmarks, CAI mainly manifests as instability in the tibiotalar joint, while the subtalar joint is affected to some extent but not as significantly. As the first joint to bear the impact of ground contact, the subtalar joint relies heavily on the stability provided by the lateral, medial, anterior, and posterior talocalcaneal ligaments.

During walking in humans, both feet alternate weight bearing, so an abnormality in one foot can affect the posture and balance of the entire body. Both the magnitude of the vGRF and the trajectory of the COP in this study may partially explain this phenomenon. It was revealed that CAI patients exhibited a greater peak vGRF, with a significantly shorter time to peak and a greater loading rate (Figure 7a). There may be two reasons for this result. First, to compensate for ankle instability caused by damage to elastic tissues (ligaments), CAI patients may adopt a more rigid walking strategy (Read et al., 2017). Second, a lateral shift in the body’s center of gravity may occur, as evidenced by the COP trajectory trending toward the lateral side. However, due to the lack of full-body motion data, direct evidence to support this hypothesis of a shift in the center of gravity is limited. Considering the impact of walking speed on vGRF, statistical analysis within the narrow range of walking speeds (1.25 m/s to 1.76 m/s) showed no significant correlation between the peak vGRF and walking speed (Pearson correlation coefficient r < 0.3, P > 0.05). This eliminates the impact of walking speed on ground reaction forces. A greater vGRF in CAI patients indicates altered gait mechanics, reflecting an increased load on the lower limb joints, which may increase the risk of reinjury to the ankle joint. Studies by Bigouette et al. and Read et al. have both reported that CAI patients exhibit greater vGRF during the stance phase (Bigouette et al., 2016; Read et al., 2017), which is consistent with our findings. Furthermore, based on the force characteristics and balance of the entire foot, an increase in Fr can lead to an increase in ankle dorsiflexor force (Fm) (Figure 7b). This is based on equation R × Fr = r × Fm proposed by Carrier et al. where R is the moment arm of the ground reaction force, and r is the moment arm of the ankle plantarflexor force (Carrier et al., 1994). This finding indicates that CAI not only affects the kinematics of the AJC but also alters the forces acting on the AJC. Among the 15 patients, 11 showed a more lateral trajectory of the COP under the foot, which may be associated with impaired coordination and balance during walking caused by CAI (Wan et al., 2023). The other 4 individuals, possibly due to milder symptoms, did not exhibit significant deviation. The lateral longitudinal arch of the foot is relatively flat, and when analyzed from this perspective, the shift of the COP toward the lateral side can provide a relatively stable interface for the unstable foot of CAI patients. This shift could be a temporary self-protection mechanism during walking, potentially alleviating ankle pain or joint surface wear. However, it does not prevent the damage from progressively worsening. Wikstrom et al. proposed that there are differences in body posture control between patients with CAI and healthy individuals (Wikstrom et al., 2010), and Hass et al. suggested that CAI alters sensorimotor function at the spinal level, potentially by modifying central control mechanisms (Hass et al., 2010). Our findings showed that the trajectory of the COP under the foot in CAI patients was more lateral than that in controls (Figure 7c). This result suggested that CAI may affect the movement of the entire lower limb and potentially impacts the spine and upper body, thereby altering balance or coordination during walking. However, this study lacks full-body motion data. Future research needs to employ more comprehensive and effective methods to validate these conclusions.

This study has several limitations. First, although both males and females were included in the participant group, the sample size was not large enough to compare the kinematic or mechanical differences between the sexes. Previous studies have shown a greater incidence of ankle sprains in women (Mason et al., 2022) and sex-related differences in the velocity of COP movement (Saarinen et al., 2022). Future research should increase the sample size to identify these sex differences. Second, this study focused on individuals with unilateral ankle instability due to ligament injuries and compared them to healthy participants. The unaffected side of the patients was not considered, which may also exhibit differences in joint motion and GRF compared to the affected side. This is another area worth investigating in future studies. Due to the limited imaging area of the biplanar radiography system, only the stance phase of the gait could be captured, and images of the ankle joint during the swing phase were not obtainable. Additionally, this study did not explore potential influences of neuromuscular control differences (such as muscle activation patterns) in CAI patients, which could be further investigated in the future by integrating muscle or neurological testing equipment.

In conclusion, this study proposed that CAI patients exhibit not only altered 3D motion and stability of local joints (i.e., the AJC) but also altered vGRF and COP distributions. This study provides critical insights into the altered kinematics of the AJC and gait mechanics in CAI patients. By deepening the understanding of these biomechanical changes, clinical practice and rehabilitation strategies can be better guided. This will provide effective comparative data for the clinical diagnosis of CAI and the assessment of postoperative rehabilitation.

## Data Availability

The original contributions presented in the study are included in the article/Supplementary Material, further inquiries can be directed to the corresponding authors.
