# Supplementary material for: New insights into chronic ankle instability: an in vivo evaluation of three-dimensional motion and stability of the ankle joint complex
Source: Front Bioeng Biotechnol. 2025 Mar 26;13:1556291. doi: 10.3389/fbioe.2025.1556291 (PMC11979236; doi:10.3389/fbioe.2025.1556291)
Supplement: Supplementary file 1 [file DataSheet1.docx]

Supplementary Material

# Supplementary Tables

**Table S1.** Demographic characteristics and radiographic grading of the degree of ligamentous injury.

|  | CAI (n=15) | Control (n=15) |
| --- | --- | --- |
| Age (yr) | 35.5±6.5 | 33.5±7.5 |
| Height (cm) | 165±3.8 | 168±4.2 |
| Weight (Kg) | 68.5±10.2 | 74.5±12.6 |
| BMI (Kg/m^2^) | 21.6±3.5 | 22.3±4.2 |
| Sex (male, female) | 6, 9 | 8, 7 |
| Side (right, left) | 7, 8 | 7, 8 |
| ATFL (0, I, II, III) | 0, 2, 6, 7 | -- |
| PTFL (0, I, II, III) | 9, 3, 3, 0 | -- |
| CFL (0, I, II, III) | 0, 4, 8, 3 | -- |
| Time since the initial sprain (months) | 24.6±7.2 | -- |
| Number of ankle sprains | 6.8±5.2 | -- |

# Supplementary Figures


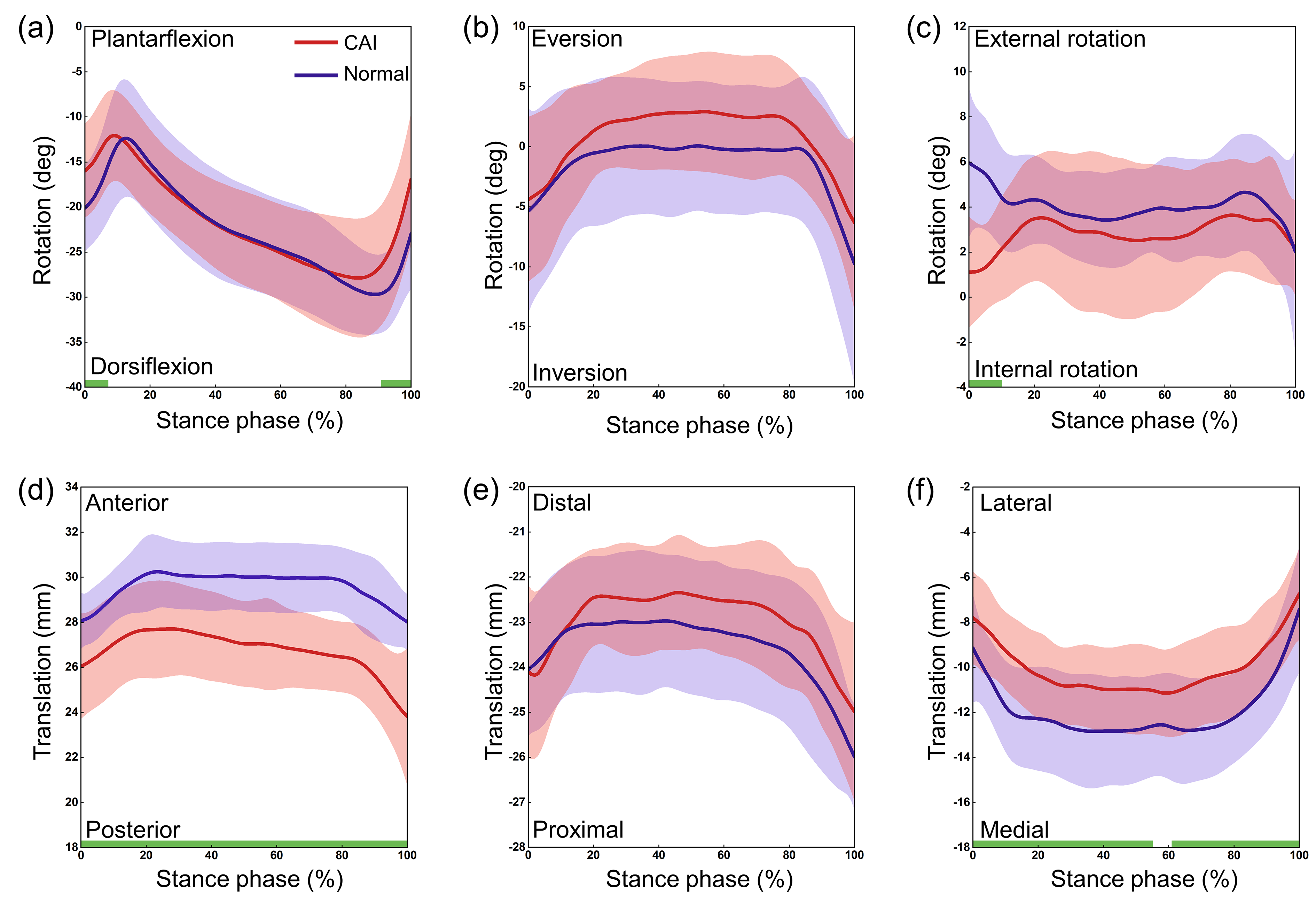


**Figure S1.** 3D kinematics of the tibio-calcaneal joint during the stance phase of gait in the CAI group (red) and control group (blue). The shaded areas represent ± 1 standard deviation. The time intervals with significant differences are marked with thick green lines below each subplot.
